# Torsion of the Epididymal Appendage in a Child

**DOI:** 10.7759/cureus.13412

**Published:** 2021-02-18

**Authors:** Ahmad Kharsa, Florentino Saenz Rios, Quan D Nguyen

**Affiliations:** 1 Department of Radiology, University of Texas Medical Branch, Galveston, USA

**Keywords:** appendage, appendix, epididymis, scrotal ultrasound, torsion, colored flow doppler ultrasound

## Abstract

The epididymal appendage, or the appendix epididymis, is a developmental remnant of the mesonephric duct that sprouts from the head of the epididymis. Due to its pedunculated anatomical configuration, the epididymal appendage is prone to torsion and can become a rare cause of an acute scrotum. Given its often challenging and atypical presentation, high clinical suspicion and Doppler ultrasonography are needed to guide diagnosis. We report a challenging case of epididymal appendage torsion that required prompt surgical exploration to accurately diagnose. Awareness of this condition and its radiological findings can play an important role in reaching an accurate diagnosis and avoiding surgical intervention.

## Introduction

Torsion of the intrascrotal appendages is responsible for approximately 45%-60% of common urologic emergencies within the pediatric population. Of specific interest, the epididymal appendage, or the appendix epididymis, is a developmental remnant of the mesonephric/Wolfian duct that sprouts from the head of the epididymis with an autopsy incidence of 20%-24%. Although such a structure is generally of not much clinical significance, its pedunculated configuration makes it prone to torsion and a rare cause of an acute scrotum [1]. While torsion of the intrascrotal appendages rarely requires surgical intervention, its atypical clinical presentation often makes it challenging to distinguish from testicular torsion, a more serious diagnosis that often requires prompt surgical exploration. Herein, we present a challenging case of epididymal appendage torsion that required prompt surgical exploration to accurately diagnose.

## Case presentation

**Figure 1:**
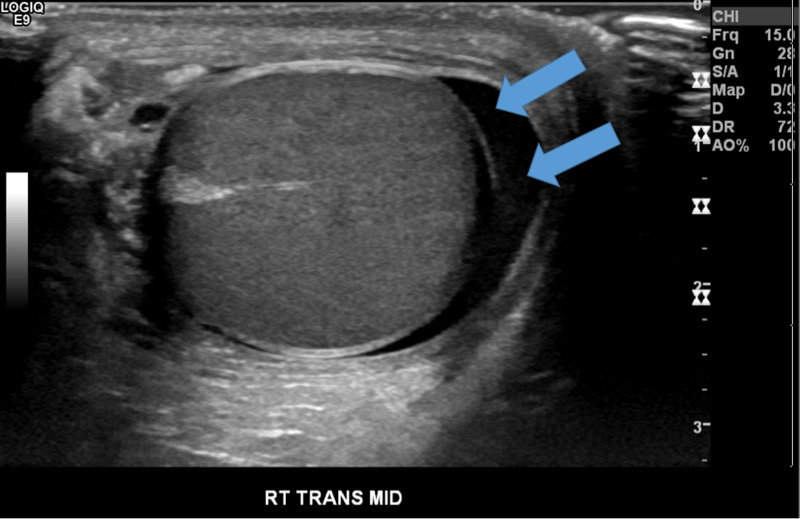
Transverse grey-scale sonography of the right testicle Double blue arrows: small volume reactive hydrocele surrounding the right testicle.

An otherwise healthy 14-year-old presented to the emergency room with right-sided scrotal pain. The pain developed gradually and was constant in frequency over the last 5-6 days. However, in the last 24 hours prior to presentation, the patient reported significant worsening of the pain associated with new onset of testicular swelling. No history of trauma, dysuria, or fever was reported. Upon physical examination, testes were descended bilaterally. The right testicle was mildly tender and indurated coupled with an exquisitely tender right epididymis. Prehn's sign was negative. The left testicle exhibited normal findings. No masses were palpated and a positive cremasteric reflex was observed bilaterally. Grey-scale sonographic examination of the scrotum was performed, revealing a small volume reactive hydrocele surrounding the right testicle (Figure 1).

**Figure 2:**
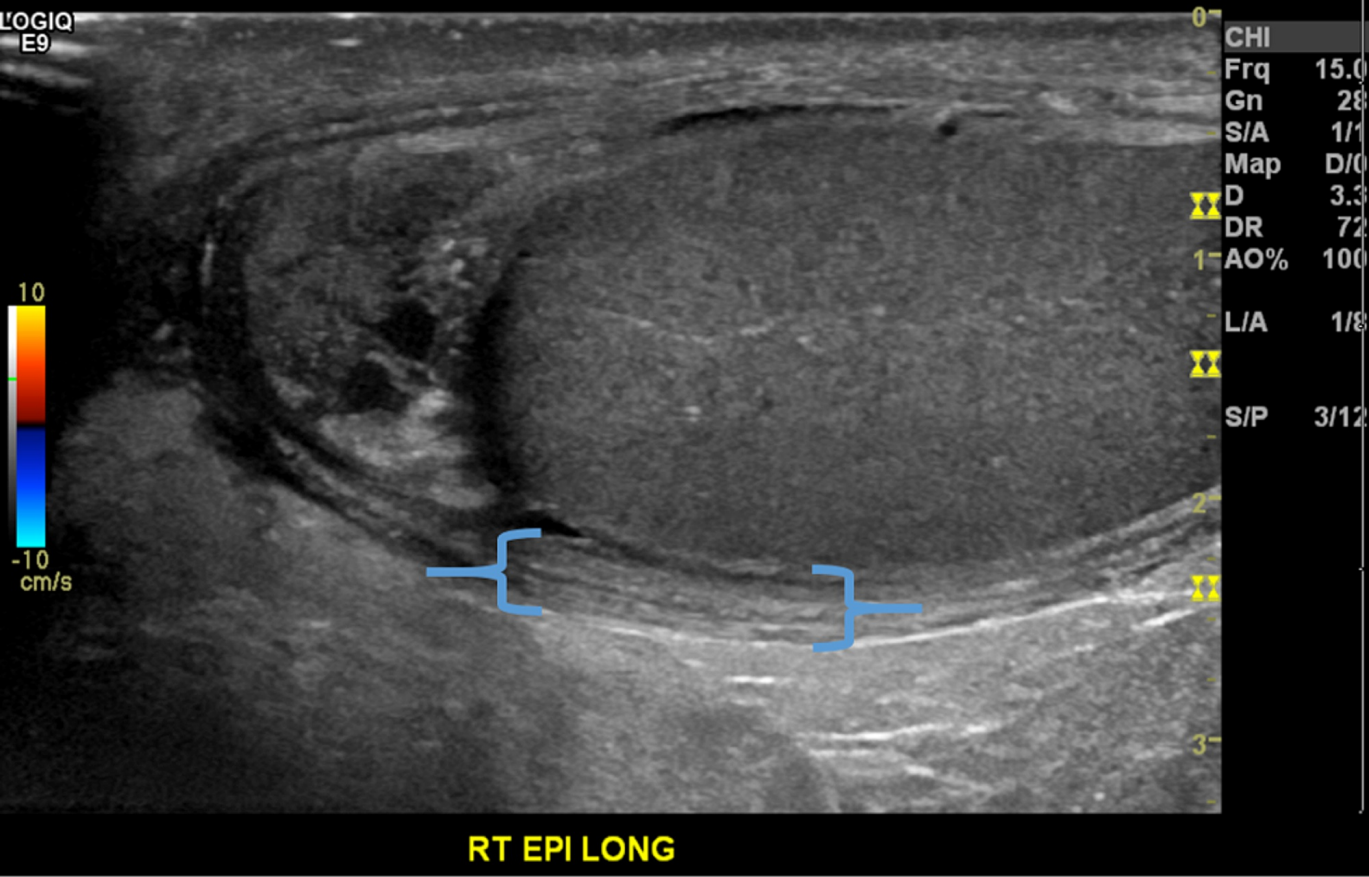
Long grey-scale ultrasonography of the right hemi-scrotum Blue brackets: significant scrotal edema

**Figure 3:**
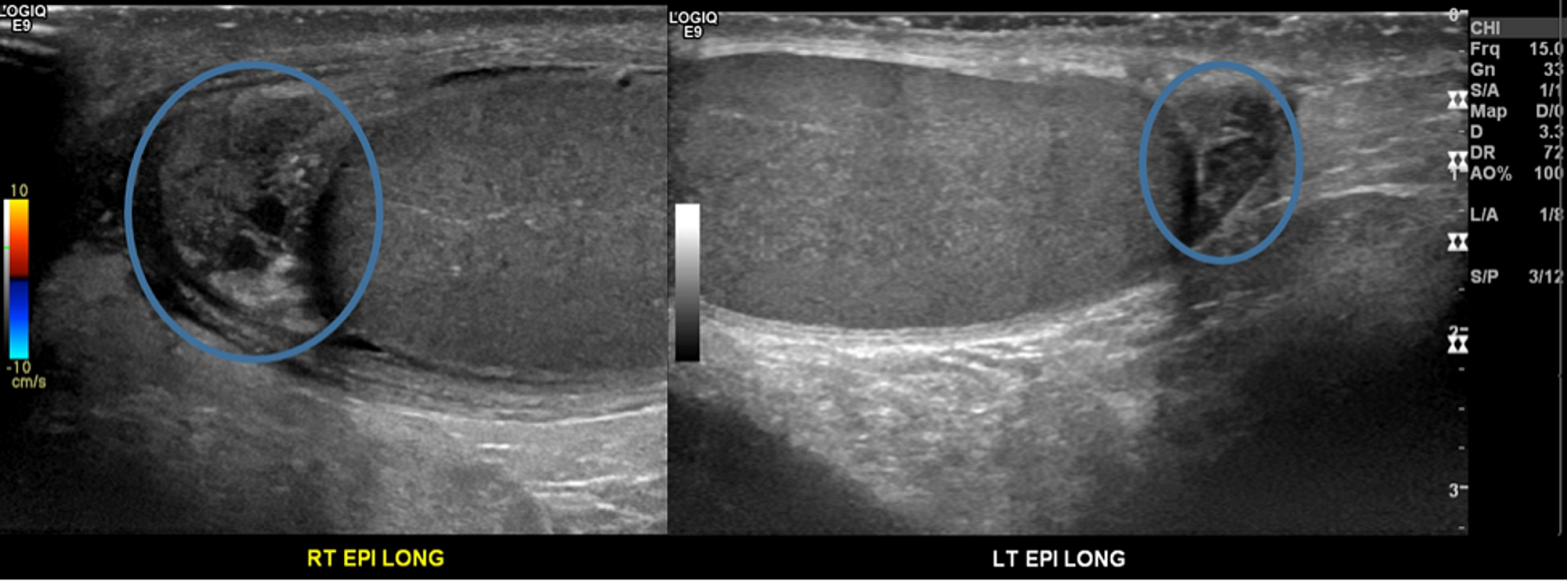
Long grey-scale ultrasonography of the bilateral epididymis Blue circles: slightly edematous right epididymis measuring 15 x 10 x 10 mm compared to the left epididymis measuring at 9 x 7 x 9 mm.

In addition, signs of significant edema were remarkable within the right scrotal wall and right epididymis, as compared to their left counterparts (Figures 2-3).

**Figure 4:**
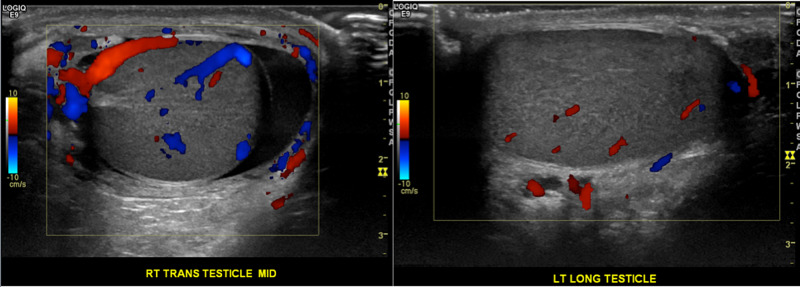
Color Doppler ultrasonography of the bilateral testes Doppler ultrasonography demonstrates normal color flow across both testes.

**Figure 5:**
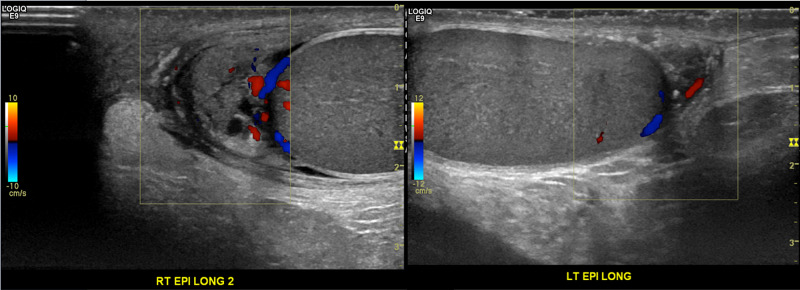
Doppler ultrasonography of the bilateral epididymides Doppler ultrasonography revealed normal vascularity across the bilateral epididymides.

**Figure 6:**
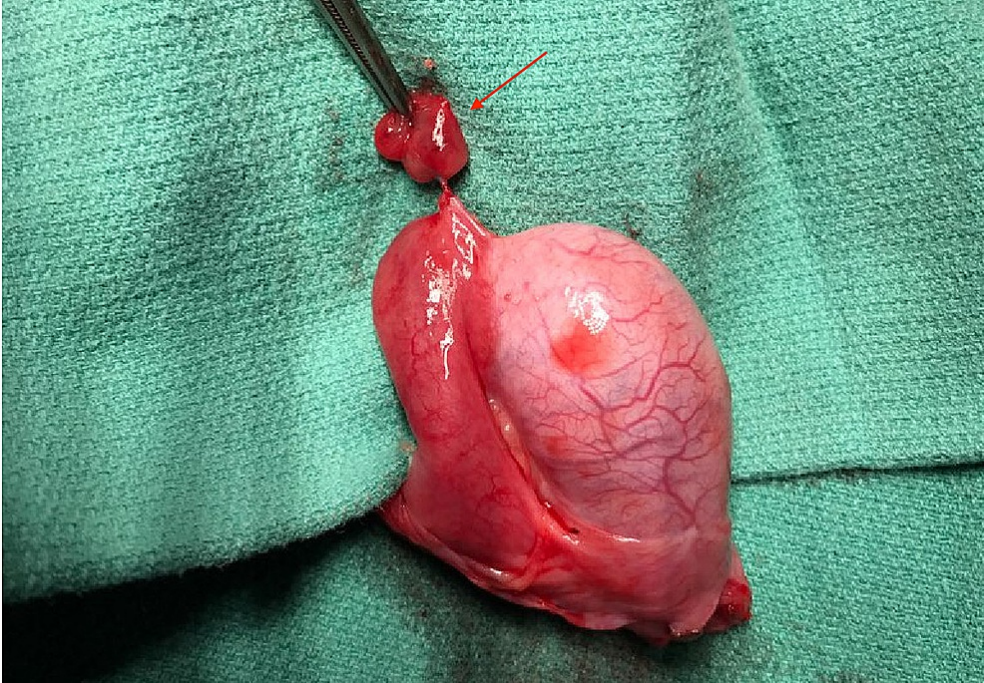
Gross pathological specimen of the torsed epididymal appendage Red arrow: Torsed epididymal appendage.

Doppler ultrasonography revealed normal vascularity across both testes and the epididymides (Figures 4-5). Given these findings, there was a radiological concern for the presence of vascular artifact, which may falsely exclude a diagnosis of testicular torsion. As such, the patient was sent to the operating room for emergent scrotal exploration. A torsed epididymal appendix was discovered and was subsequently excised (Figure 6). Finally, the patient tolerated the uncomplicated procedure well and was medically stable to discharge home. A two-week follow-up with the patient revealed no postoperative complications. 

## Discussion

Torsion of the epididymal appendage classically presents in children aged 7-14 years and rarely occurs in adulthood [2]. Patients classically present with gradual onset of scrotal pain in the presence of a preserved cremasteric reflex, which may be associated with scrotal swelling and erythema. Specifically, the presence of a cremasteric reflex is of significant clinical value in ruling out testicular torsion. In addition, patients may also have other nonspecific symptoms such as nausea, vomiting, or abdominal pain. Furthermore, while only present in about 20% of cases, the palpation of a paratesticular nodule at the superior aspect of the testis, a so-called “blue-dot” sign, is pathognomonic for this condition. The combination of the blue-dot sign with clear palpation of an underlying normal, non-tender testis allows for the exclusion of testicular torsion on clinical grounds alone [1,2]. Nevertheless, the clinical picture for a torsed epididymal appendage can often be misdiagnosed as epididymitis or epididymoorchitis, making an assessment with sonography crucial to discerning the ambiguity. 

On grey-scale ultrasonography, the torsed appendage usually appears as an enlarged nodular or oval mass, with variable echogenicity [2]. While the detection of an epididymal appendage is not pathognomonic for torsion, its detection with a diameter greater than 5.6 mm is highly suggestive of torsion, with almost 90% sensitivity [2,3]. In the event of infarction of the torsed peduncle, the appendage may detach and wander inside the scrotum, appearing as minute mobile particles. Findings consistent with a thickened scrotal wall and a reactive hydrocele may also be present. However, these findings are non-specific and may be found in individuals with epididymitis. Epididymal tumors may also mimic epididymal appendage torsion but the absence of mass-like lesions and vascular flow in the epididymis on the scrotal ultrasound distinguishes torsion from an epididymal tumor [3].

While scrotal scintigraphy would reveal diffusely enhanced activity in the affected hemiscrotum, such findings are not specific enough and are also seen in the case of epididymitis [1]. Further evaluation with Doppler ultrasonography would reveal an avascular appendage associated with significant hypervascularity in the surrounding tissues, especially the epididymis and testis [3]. If present, this hyperemia would highlight the darkened avascular appendix against its colorful background, providing a valuable finding in the correct diagnosis of this condition [2]. Nevertheless, this surrounding hyperemia is not always present and ranges in incidence from 30% to 90% [3]. Furthermore, it is to be noted that an improperly increased color gain may result in a “bleeding artifact” and obscure an avascular torsed epididymal appendix. Similarly, a delay in performing Doppler ultrasonography may lead to increased vascular flow in the epididymis. This may lead to an erroneous diagnosis of epididymitis and unnecessary antibiotic treatment [2,3].

Accurate and rapid evaluation of the acute scrotum is critical for patient management. As opposed to the necessary surgical intervention for a spermatic cord torsion, conservative non-surgical management can be utilized in the setting of a torsed intrascrotal appendage as it is self-limited. As such, management is mainly focused on pain management with nonsteroidal anti-inflammatory drugs (NSAIDs), ice packs, and scrotal elevation. Nevertheless, surgical intervention through appendage excision can shorten the average duration of symptoms [1,4].

## Conclusions

Torsion of the epididymal appendage is an uncommon cause of an acute scrotum in the pediatric population. Given its often challenging and atypical presentation, high clinical suspicion and Doppler ultrasonography are needed to guide diagnosis. Nevertheless, in clinically equivocal cases, prompt surgical exploration is necessary to reach a clear diagnosis.
